# The challenge of treating hepatitis C virus infection in children with comorbidities

**DOI:** 10.1007/s00431-025-06038-3

**Published:** 2025-03-10

**Authors:** Engy Adel Mogahed, Nevian Nabil, Haytham Ghita, Afaf Enayet, Hanaa El-Karaksy

**Affiliations:** 1 Pediatric Hepatology Unit, Department of Pediatrics, Cairo University, Cairo, Egypt; 2 2 B Sama City, Katamya, Cairo, 11439 Egypt

**Keywords:** Children, Comorbidities, DAAs, Direct-acting antivirals, Efficacy, HCV, Safety, Sustained virologic response, SVR

## Abstract

Direct-acting antivirals (DAAs) have revolutionized hepatitis C virus (HCV) treatment and enabled the treatment of those who could not be treated using interferon. The aim of this work was to assess the efficacy and safety of oral DAAs in HCV-infected children with associated comorbidities. This analytical retrospective study included children with HCV mono-infection versus those with associated comorbidities. The study included 187 HCV-infected children aged 6–18 years; 114 patients (61%) had associated comorbidities. The most frequent comorbidities were hematological disorders (30.7%), followed by renal and cardiac diseases. Baseline total bilirubin, aspartate aminotransferase, and gamma glutamyl transpeptidase were significantly more elevated in patients with comorbidities. Sustained virologic response (SVR) was achieved in 100% of patients with HCV mono-infection versus 98.2% of patients with comorbidities. The most frequently reported treatment adverse effects were headache, asthenia, and irritability. All side effects were transient and did not necessitate treatment discontinuation.

*Conclusion*: DAAs allowed treatment of HCV-infected children with comorbidities with high SVR and excellent safety profile. Treatment with sofosbuvir/ledipasvir achieved an SVR of 98.9% in HCV-infected children with comorbidities. Treatment was safe and well tolerated with mild transient adverse events.

**Table:** 

**What is Known**: • *The novel DAAs have revolutionized the landscape of HCV treatment and enabled the treatment of those who could not be treated using IFN.* • *When treating HCV, clinicians should take into consideration the presence of other comorbid conditions.* *In the IFN-RBV era, many HCV patients with comorbidities were ineligible for therapy*.	
**What is New**: • *There are limited data in the literature about the efficacy and tolerability of DAAs in children with comorbidities.* • *We reported in the current study that DAAs allowed treatment of HCV-infected children with comorbidities with high SVR and excellent safety profile. These patients should be offered treatment with oral DAAs to help decrease the infectious pool and hence reach the ambitious final goal of global eradication.*

**What is Known**:

• *The novel DAAs have revolutionized the landscape of HCV treatment and enabled the treatment of those who could not be treated using IFN.*

• *When treating HCV, clinicians should take into consideration the presence of other comorbid conditions.* *In the IFN-RBV era, many HCV patients with comorbidities were ineligible for therapy*.

**What is New**:

• *There are limited data in the literature about the efficacy and tolerability of DAAs in children with comorbidities.*

• *We reported in the current study that DAAs allowed treatment of HCV-infected children with comorbidities with high SVR and excellent safety profile. These patients should be offered treatment with oral DAAs to help decrease the infectious pool and hence reach the ambitious final goal of global eradication.*

## Introduction

Hepatitis C virus (HCV) infection in children continues to be a serious public health problem, with an estimated global prevalence equating to 3.26 million viremic children worldwide [1]. HCV antibodies were detected in 0.38% of Egyptian adolescents of whom 78.7% had positive HCV RNA [2].

When treating HCV, clinicians should take into consideration the presence of other comorbid conditions. In the interferon-ribavirin (IFN-RBV) era, many HCV patients with comorbidities were ineligible for therapy. IFN-RBV regimen was contraindicated in many neuropsychiatric conditions and cardiovascular and autoimmune diseases. It was estimated that around 20% of patients with HCV were not able to receive IFN, which left a big burden of untreated patients [3]. Moreover, it has been proved that such comorbidities might reduce the response rate to IFN-RBV therapy with a higher frequency of treatment-related adverse events [4]. The presence of other comorbid conditions may lead to more progressive liver disease [5]. Comorbidities such as hematological illnesses with iron overload, obesity, and malignancies may hasten the development of hepatic fibrosis in children and adolescents [6].

The novel direct-acting antivirals (DAAs) have revolutionized the landscape of HCV treatment and enabled the treatment of those who could not be treated using IFN [7]. DAAs show a high safety profile and are administered for short periods. These factors allowed the treatment of special patient populations as patients with comorbidities, who were previously known to be difficult to treat [4]. However, a considerable portion of patients with HCV and comorbidities receive multiple medications with variable pharmacokinetics and pharmacodynamics that can affect DAAs. Dealing with these potential drug–drug interactions adds another challenge in treating these patients as some drugs may reduce the concentrations of DAAs with a risk of virological failure. Therefore, drug–drug interactions need to be considered to optimize the pharmacotherapeutic outcome of DAAs [7].

There are limited data in the literature about the efficacy and tolerability of DAAs in children with comorbidities. We aimed in this study to assess the safety and efficacy of oral DAAs in HCV-infected children and adolescents with associated comorbidities. In addition, our secondary aim was to investigate the effect of these comorbidities on severity of liver disease.

## Patients and methods

### Study design

This single-center retrospective analytical study included HCV children and adolescents who were treated with oral DAAs. The study was conducted at the Pediatric Hepatology Unit, Cairo University Pediatric Hospital, Cairo, Egypt. The study was conducted in accordance to the Declaration of Helsinki, and the study protocol was approved by Cairo University institutional review board and ethical committee. The informed consent was waived because of the retrospective nature of the study and that no personal identifiable data will be processed or published.

### Patients

The study included children and adolescents with chronic genotype 4 HCV (considering that genotype 4 is almost the exclusive genotype prevalent in Egyptian population [8]). Recruited children included both sexes, 6–18 years old who presented to the Pediatric Hepatology Unit during the time period from 2017 to 2022. It included all children with chronic HCV either treatment naïve or IFN experienced, patients with different comorbidities or HCV mono-infected, and patients with extrahepatic manifestations of HCV. Patients were excluded from treatment if they had insufficient data in their files. Patients with a history of hepatitis B virus infection, or patients with significant or unstable cardiac disease or uncontrolled cardiac arrhythmias were not qualified for treatment.

## Methods

The following data were retrieved from patients’ files:

- Demographic data (age and sex).
- Detailed medical history including symptoms suggestive of hepatic affection and symptoms of other systems.
- Risk factors of HCV acquisition (e.g., maternal HCV, intrafamilial cases, hospitalization, operation, chemotherapy, blood product transfusion).
- Previous or current associated medical or surgical condition.
- Detailed medication history (including previous HCV treatment and current therapy for comorbidities).
- General, systemic, and abdominal examination.

Results of baseline (pre-treatment) investigations recruited from patients’ files included complete blood count (CBC), total and direct serum bilirubin, alanine aminotransferase (ALT), aspartate aminotransferase (AST), alkaline phosphatase (AP), gamma glutamyl transpeptidase (GGT), serum albumin, international normalized ratio (INR), serum creatinine, 12-lead (electrocardiogram) ECG, echocardiography and Holter ECG (for cardiac and renal patients), HCV viral load, and fibrosis stage using Fibroscan (FibroScan Echosens, Paris, France). Degree of liver fibrosis was categorized into no fibrosis (F0), mild (F0–F1, F1), moderate (F1–2, F2), and marked (F3, F4). The results were expressed in kilopascals (kPa). F0: 0–5.4 kPa, F1: 5.5–7.1 kPa, F1–F2: 7.2–8.7 kPa, F2: 8.8–9.5 kPa, F3: 9.6–12.5 kPa, F3–F4: 12.6–14.5 kPa, and F4: >14.5 kPa.

Data related to treatment with oral DAAs were collected.

1. The only approved DAA available for children in Egypt was sofosbuvir/ledipasvir (SOF/LED) (either original Harvoni or the generic form [ledisbuvir]). Children aged ≥ 12 years or weighing ≥ 35 kg received 400/90 mg SOF/LED as a single tablet once daily orally for 12 weeks. Children < 12 years and weighing 17–35 kg received 200/45 mg SOF/LED as a single half tablet once daily orally for 12 weeks.
2. For patients with associated chronic diseases who were receiving other medications, DDIs were checked prior to DAA initiation. Medications which had an effect on DAAs’ absorption were properly spaced or discontinued during the 3 months of DAAs therapy (e.g., proton pump inhibitors and calcium containing preparations).
3. Data of potential treatment adverse events during the course of treatment recorded by the caregivers or the examining physicians such as jaundice, asthenia, fatigue, headache, cough, dyspnea, nausea, vomiting, diarrhea, insomnia, irritability, skin rash, dizziness, arrhythmia, hyperlipidemia, and renal dysfunction.
4. Laboratory data of follow-up visits at weeks 4, 12, and 24 of therapy: liver functions and HCV RNA to detect early virologic response (EVR), end-of-treatment response (ETR), and sustained virologic response (SVR). Treatment efficacy was achieved if the patient had SVR (negative HCV RNA 12 weeks after treatment discontinuation).

Patients with HCV and comorbidities were compared to patients with HCV mono-infection as regards demographic data, risk factors of HCV acquisition, baseline and end-of-treatment liver functions, and baseline viral load and degree of hepatic fibrosis. In order to assess the efficacy and safety of HCV treatment with oral DAAs, both groups were compared as regards treatment response (SVR) and frequency of treatment adverse effects.

### Statistical methods

Continuous variables as age and laboratory results were expressed as mean ± standard deviation (SD) or median with interquartile range (IQR) of 25–75%. Categorical variables such as comorbidities, risk factors, growth parameters, and adverse effects were expressed as absolute frequencies and percentage. Group comparisons were performed using Student’s *t*-test or Mann–Whitney *U* test and Pearson chi-square or Fisher exact test for continuous and categorical variables, respectively. Correlation was evaluated by the Spearman correlation coefficient. All statistical analyses were performed by SPSS Statistics version 21. All *P* values were two sided, and *P* values < 0.05 were considered statistically significant.

## Results

The present study included 187 chronically infected HCV children and adolescents aged 6–18 years; 123 were males (65.8%). Fifteen patients (8%) were non-responders to previous course of INF-RBV. IFN was discontinued for at least 1 year before starting DAAs.

Sixty-one percent of the patients (114/187) had comorbid conditions other than HCV infection, a quarter of whom had more than one comorbidity (29/114; 25.4%). The most frequently associated comorbidities were hematological disorders in 35/114 patients (30.7%), followed by renal diseases (either on hemodialysis or not) in 22/114 (19.3%) and congenital heart diseases in 19/114 (16.7%) (Table 1). One patient had lichen planus as an extra-hepatic manifestation of HCV.

**Table 1:** Associated comorbid conditions in 114 hepatitis C virus–infected children and adolescents

Associated comorbidity	Number of patients	%
Hematological diseases	35	30.7
Renal diseases	22	19.3
Congenital heart diseases	19	16.7
Rheumatological diseases	18	15.8
Previously treated oncological conditions	15	13.2
Neurological conditions	12	10.5
Post–solid organ and hematopoietic stem-cell transplantation	8	7
Hepatic diseases other than HCV	7	6.1
Genetic disorders	7	6.1
Gastrointestinal disorders	5	4.4
Endocrinal diseases	4	3.5
Bronchial asthma	1	0.9
Patients with more than one comorbidity	29	25.4

Table 2 shows the demographic data and risk factors for HCV acquisition in children with and without associated diseases. Age and gender were comparable between both groups. History of maternal HCV and other intrafamilial cases with HCV was significantly more frequent in patients with HCV mono-infection (*P* < 0.001), while history of hospitalization and blood product transfusion were more significantly reported in patients with comorbidities (*P* < 0.001). Seventy-eight percent of patients with comorbidities were receiving other therapies during the period of HCV treatment.

**Table 2:** Comparison of demographics and risk factors of HCV acquisition in children mono-infected with HCV and children with comorbidities

Variable	Patients with HCV mono-infection (*N* = 73)	Patients with comorbidities (*N* = 114)	*P* value
Age in years; mean ± SD	11.05 ± 2.3	11 ± 3.3	0.9
Range	6–17	6–18	
Sex			
Male: *n* (%)	54 (61.6)	69 (60.5)	1.0
Female: *n* (%)	28 (38.4)	45 (39.5)	
Risk factors for HCV acquisition: *n* (%)			
Maternal HCV	41 (56.2)	10 (8.8)	< 0.001*
Intrafamilial cases	36 (49.3)	26 (22.8)	< 0.001*
Blood product transfusion	10 (13.7)	87 (76.3)	< 0.001*
Hospitalization	43 (58.9)	106 (93)	< 0.001*
Previous operations	35 (47.9)	67 (58.8)	0.2
Dental procedure	33 (45.2)	35 (30.7)	0.06
Interferon-ribavirin experienced patients: *n* (%)	8 (11)	7 (6.2)	0.3

*HCV* hepatitis C virus

^*^P value is significant

Clinical examination of the patients revealed that stunted growth and organomegaly were significantly more frequent among patients with comorbidities. Baseline investigations revealed that total bilirubin, AST, and GGT were significantly more elevated and synthetic functions were more affected in patients with comorbidities (Table 3). HCV viral load was comparable in both groups. Although patients with marked degrees of fibrosis were more frequent in patients with comorbidities, the difference was not statistically significant (Table 3).

**Table 3:** Comparison of baseline clinical findings, liver functions, HCV viral load, and hepatic fibrosis in children mono-infected with HCV and children with comorbidities

Variable	Patients with HCV mono-infection (*N* = 73)	Patients with comorbidities (*N* = 114)	*P* value
Weight percentile: number of patients (%)			0.2
– Normal	67 (91.8)	88 (77.2)	
– Underweight	5 (6.8)	25 (21.9)	
– Overweight	1 (1.4)	1 (0.9)	
Height percentile: number of patients (%)			< 0.001*
– Normal	70 (95.9)	78 (68.4)	
– Stunted	3 (4.1)	36 (31.6)	
Hepatomegaly: number of patients (%)	1 (1.4)	22 (19.3)	< 0.001*
Splenomegaly: number of patients (%)	0 (0)	18 (15.8)	< 0.001*
Total bilirubin (up to 1 mg/dl):			0.01*
– Median (IQR)	0.35 (0.3–0.6)	0.5 (0.3–0.7)	
– Range	0.1–1.33	0–5.6	
Direct bilirubin (mg/dL):			0.7
– Median (IQR)	0.1 (0.1–0.2)	0.1 (0.1–0.2)	
– Range	0–0.8	0–1.6	
ALT (up to 40 U/L):			0.2
– Median (IQR)	53.5 (34–83)	57.5 (35–109)	
– Range	8–245	5–750	
AST (up to 50 U/L):			0.01*
– Median (IQR)	43 (33–60)	56 (36–78.5)	
– Range	6–266	9–389	
Number of patients with elevated ALT and/or AST (%)	73 (71.2)	114 (78.1)	0.3
AP (up to 360 U/L):			0.9
– Median (IQR)	219 (152–292)	225 (154–301)	
– Range	18–972	66–862	
GGT (up to 40 U/L):			< 0.001*
– Median (IQR)	30 (19–50)	52.5 (25–115)	
– Range	4–177	3–954	
Albumin (3.5–5 gm/dL):			0.02*
– Mean ± SD	4.3 ± 0.4	4.1 ± 0.6	
– Range	3.2–5.2	1.3–5.5	
INR:			0.02*
– Mean ± SD	1.05 ± 0.09	1.08 ± 0.1	
– Range	0.9–1.6	0.9–1.6	
HCV RNA (up to 15 IU/mL): – Median (IQR)	4.22 × 10^9^ (6.995 × 10^8^–1.72 × 10^9^)	3.935 × 10^9^ (6.916725 × 10^4^–1.03 × 10^9^)	0.5
– Range	1.92 × 10^5^–2.86 × 10^7^	441–4.28 × 10^7^	
Liver fibrosis degree by Fibroscan: (*N* = 185)^#^			0.1
– No	53 (72.6)	66 (58.9)	
– Mild	13 (17.8)	23 (20.5)	
– Moderate	4 (5.5)	8 (7.1)	
– Marked	3 (4.1)	15 (13.4)	

*ALT* alanine aminotransferase, *AP* alkaline phosphatase, *AST* aspartate aminotransferase, *GGT* gamma glutamyl transpeptidase, *HCV RNA* hepatitis C virus ribonucleic acid, *INR* international normalized ratio, *IQR* interquartile range, *SD* standard deviation

*P value is significant

^#^Fibroscan could not be performed for 2 of the patients with comorbidities (end-stage renal disease) due to massive ascites

Results of EVR were available for 162 patients without a statistically significant difference in both groups. All patients achieved ETR. SVR was achieved in 100% of patients with HCV mono-infection and in 98.2% of patients with comorbidities without a statistical difference (Table 4). The two patients who failed to achieve SVR had end-stage renal disease (ESRD) and were on regular hemodialysis. Although these two patients had negative HCV RNA at end of treatment, they showed positive HCV RNA 12 weeks after treatment discontinuation with the possibility of either breakthrough of re-infection. The patient with lichen planus showed marked improvement of his skin lesions with treatment.

**Table 4:** Comparison of treatment response and liver functions at end of treatment in children mono-infected with HCV and children with comorbidities

Variable	Patients with HCV mono-infection (*N* = 73)	Patients with comorbidities (*N* = 114)	*P* value
Early virologic response (in 162 patients): number of patients (%)	62/64 (96.9)	96/98 (98)	0.6
End-of-treatment response: number of patients (%)	73 (100)	114 (100)	NA
Sustained virologic response: number of patients (%)	73 (100)	112 (98.2)	0.5
Total bilirubin (up to 1 mg/dL): – Median (IQR) – Range	0.4 (0.3–0.5) 0.1–1.7	0.4 (0.3–0.7) 0.1–4.7	0.1
Direct bilirubin (mg/dL): – Median (IQR) – Range	0.1 (0.1–0.1) 0–1.0	0.1 (0.1–0.2) 0–3.5	0.02*
ALT (up to 40 U/L): – Median (IQR) – Range	21 (17–29.75) 9–130	22.5 (14–33) 6–226	0.9
AST (up to 50 U/L): – Median (IQR) – Range	26 (22–30.25) 13–157	26 (20–35.5) 10–227	0.7
Number of patients with elevated ALT and/or AST at end of treatment (%)	8 (11)	19 (16.7)	0.4
AP (up to 360 U/L):			
– Median (IQR)	225 (168–288)	198 (142–282)	0.1
– Range	62–450	60–893	
GGT (up to 40 U/L):			0.009*
– Median (IQR)	18 (13–21)	22.5 (15–35.7)	
– Range	2–74	1.54–347	
Albumin (3.5–5 g/dL):			0.2
– Mean ± SD	4.1 ± 0.4	3.98 ± 0.7	
– Range	3.4–5.3	1.1–5.2	
INR:			0.7
– Mean ± SD	1.1 ± 0.1	1.1 ± 0.1	
– Range	0.7–1.9	0.9–1.4	

*ALT* alanine aminotransferase, *AP* alkaline phosphatase, *AST* aspartate aminotransferase, *DAAs* direct-acting antivirals, *GGT* gamma glutamyl transpeptidase, *HCV* hepatitis C virus, *INR* international normalized ratio, *IQR* interquartile range, *NA* not applicable, *SD* standard deviation

*P value is significant

At end of treatment, ALT and/or AST were still elevated in 27 patients (14.4%), while GGT was elevated in 19 patients (10.2%). Elevated transaminases at end of treatment were not related to the presence of comorbidities (*P* = 0.4). GGT was significantly higher at end of treatment in patients with comorbidities (*P* = 0.009) (Table 4).

The most frequently reported treatment adverse events were headache, asthenia, and irritability (Table 5). There was not a statistical difference in the frequencies of side effects between patients with or without comorbidities. All side effects were transient and did not necessitate treatment discontinuation in any patient. Based on their ECG, three patients developed arrhythmia during treatment with DAAs (one patient who was previously treated for leukemia had infrequent pulsus trigemini and two patients with ESRD both had right bundle branch block). The three patients were clinically asymptomatic, and their heart rate and rhythm were repeatedly normal throughout the duration of treatment and none of them developed bradycardia. Those arrhythmias were transient, and treatment was safely completed for the three patients after cardiological consultation and follow-up.

**Table 5:** Reported side effects of oral of DAAs received in children mono-infected with HCV and children with comorbidities

Variable	Patients with HCV mono-infection (*N* = 73) *N* (%)	Patients with comorbidities (*N* = 114) *N* (%)	*P* value
Headache	34 (46.6)	43 (37.7)	0.2
Asthenia/fatigue	31 (42.5)	42 (36.8)	0.4
Irritability	26 (35.6)	44 (38.6)	0.7
Dizziness	22 (30.1)	29 (25.4)	0.5
Cough	21 (28.8)	41 (36)	0.3
Nausea	10 (13.7)	21 (18.4)	0.4
Dyspnea	10 (13.7)	20 (17.5)	0.5
Diarrhea	9 (12.3)	18 (15.8)	0.7
Insomnia	8 (11)	15 (13.2)	0.8
Skin rash	7 (9.6)	6 (5.3)	0.2
Jaundice	3 (4.1)	8 (7)	0.5
Arrhythmia	0 (0)	3 (2.6)	0.3

*DAAs* direct-acting antivirals, *HCV* hepatitis C virus

## Discussion

In the current study, we aimed to investigate the efficacy and tolerability of the currently approved DAAs among a special group of HCV-infected children and adolescents. To our knowledge, this is one of a few studies that assess treatment response in HCV-infected children with comorbidities. The Pediatric Hepatology Unit at Cairo University, Egypt, represents one of the largest specialized tertiary care centers in Egypt. That explains this relatively large number of HCV-infected children with comorbidities who presented to our center. Children with comorbidities represented 61% of the total number of treated patients with DAAs.

There is a paucity of studies in the literature that specifically targeted the population of chronic HCV-infected children with comorbidities. AbouBakr et al. [9] reported 100% SVR in their study that included 12 children with comorbidities treated with SOF/LED. The most frequently observed side effects in their study were headache, drowsiness, and fatigue. Few patients complained of nausea, vomiting, chest pain, and abdominal pain. None of the cases required drug discontinuation. In the current study, SVR was achieved in 98.9% of children with comorbidities. Most of the side effects were mild. Although three patients developed arrhythmia, it was asymptomatic and transient.

Despite the slower progression of fibrosis in HCV-infected children compared to adults, 30% of untreated children may develop worsening of fibrosis over 5 years [10]. Advanced liver disease and decompensated cirrhosis have been diagnosed in children aged as young as 3 years and as early as 1 year after infection [11]. Although higher degrees of hepatic fibrosis were more prevalent in patients with comorbidities in our study, the difference was not statistically significant. This finding agrees with Boccaccio and Bruno [12] who reported that faster disease progression was observed in HCV-infected patients with comorbidities. Moreover, we observed that failure of normalization of liver functions at end of treatment, even after HCV clearance, was more prevalent in patients with comorbidities (16.7% vs 11%). GGT was significantly elevated in patients with comorbidities who mostly receive other drugs. Elevated GGT is reported to be an indicator of drug-induced liver injury, even if not associated with an elevation of ALT and AST [13].

Children with hematological conditions represented the most common comorbidity in our cohort. In addition, we treated several cases with hematological malignancies. All these patients achieved SVR after treatment with SOF/LED. Patients with inherited blood disorders and hematological malignancies are at risk of HCV infection because of the many blood product transfusions they receive. HCV could be more aggressive in these patients secondary to iron overload and the concurrent use of hepatotoxic drugs [14, 15]. In the past, the administration of IFN-RBV therapy was restricted in these patients due to its undesirable side effects, in particular anemia [16]. Furthermore, IFN was contraindicated in patients with hematological malignancies who receive immunosuppressive therapy. Currently, these clinical challenges have largely been eliminated with the availability of the well-tolerated DAAs. In concordance with our study, a high rate of SVR was reported in multiple studies performed both on adults and children with hematological disorders and hematological malignancies who were treated with oral DAAs [15, 17, 18]. In the present study, we observed that patients with comorbidities had a significant higher frequency of jaundice and splenomegaly. This could be attributed to the high percentage of hematological disorders in our cohort including chronic hemolytic anemias.

The incidence of HCV among patients with ESRD ranges from 6 to 50% in developing countries. HCV infection has been shown to increase the risks of developing chronic kidney disease and ESRD [19], and to negatively impact the graft function and survival following renal transplant [20]. IFN was previously the standard therapy even for patients on hemodialysis; however, its SVR rate was not high, and a high rate of adverse events was observed [21, 22]. DAAs provide a new hope for this large category of patients, either by pre-transplant HCV eradication to avoid post-transplant recurrence or even in the case of post-transplantation as it is effective and well tolerated [7]. Despite the elevated plasma levels of SOF and its metabolite GS-331007 in patients with renal impairment, growing evidence supports the safety and efficacy of SOF-based regimens without any dose adjustment [23, 24]. In the current study, we have successfully treated HCV in 20 out of 22 children (SVR: 91%) with different types of renal disorders with or without hemodialysis. Another three patients were treated after kidney transplantation, and all of them achieved SVR. HCV is highly prevalent among Egyptian patients on hemodialysis [25] and those patients are subjected to the risk of re-infection after HCV treatment. Based on our results, it is worth noting that efficacy and safety of HCV treatment in children with ESRD on hemodialysis should be cautiously monitored as two of these patients did not achieve SVR (either as a breakthrough of re-infection) and another two patients had transient right bundle branch block during therapy.

In adults, DAA treatment in patients with cardiac diseases requires special precautions to avoid DDIs with antiarrhythmic drugs and statins [26]. This concern is not frequently addressed in children as their cardiac conditions are due to congenital cardiac anomalies. To ensure safety of DAAs for these patients, our patients with congenital cardiac defects (19 patients; 10.2%) were screened by 12-lead ECG and 24-h Holter ECG before and after initiation of therapy, and none of them developed arrhythmia.

Seven out of 19 patients (36.8%) with associated cardiac disease, in the present study, had high degrees of hepatic fibrosis. To assess the degree of liver fibrosis in our study, we used transient elastography (Fibroscan). The diagnostic accuracy of the Fibroscan for hepatic fibrosis has been validated in chronic viral hepatitis. However, evidence of the utility of ultrasound-based elastography for cardiac hepatopathy remains insufficient [27, 28]. As ultrasound-based elastography cannot differentiate blood from fibrosis, congestion influences the liver stiffness assessed by elastography. Thus, elastography is prone to overestimate liver fibrosis in patients with cardiac congestion [29].

The current study included 18 children with different rheumatological diseases. SVR was achieved in all these patients. Patients with rheumatoid arthritis and HCV infection are likely to have more disabling symptoms and higher activity scores [30]. Several studies concluded that HCV elimination seems to contribute to the improvement of the activity of different rheumatic diseases [31]. Moreover, management of rheumatological diseases usually requires the use of immunosuppressive agents that could potentially raise the risk of hepatotoxicity and viral flare [32]. Therefore, screening and clearance of HCV in these patients is recommended to allow optimal management.

One of the limitations in our study is the short duration of follow-up. Patients with comorbidities represent a large population and should be divided into subgroups according to their chronic disease in order to study each special population. Further long-term follow-up studies are recommended to observe rare side effects of the new DAAs on special populations and to detect the expected high risk of reinfection in the setting of horizontal transmission. In addition, in patients with comorbidities, the effect of HCV clearance on fibrosis regression and autoimmune disease activity needs to be studied. Despite these limitations, this study represents one of the largest single-center studies investigating the response of DAAs in children with comorbidities. These patients should be offered treatment with oral DAAs as they show high rate of SVR with excellent safety profile. Successful elimination of HCV from this specific group of population will help decrease the infectious pool and achieve the ambitious final goal of global eradication.

In conclusion, treatment with SOF/LED achieved SVR of 100% in children with HCV mono-infection and 98.9% in HCV-infected children with comorbidities. Treatment was safe and well tolerated with mild transient adverse events.

## Data Availability

No datasets were generated or analysed during the current study.

## Abbreviations

ALT: Alanine aminotransferase
AP: Alkaline phosphatase
AST: Aspartate aminotransferase
CBC: Complete blood count
DAAs: Direct-acting antivirals
EEG: Electrocardiogram
ESRD: End-stage renal disease
ETR: End-of-treatment response
EVR: Early virologic response
GGT: Gamma glutamyl transpeptidase
HCV: Hepatitis C virus
IFN: Interferon
INR: International normalized ratio
IQR: Interquartile range
kPa: Kilopascal
LED: Ledipasvir
RBV: Ribavirin
SOF: Sofosbuvir
SD: Standard deviation
SVR: Sustained virologic response
